# Identifying long-term and imminent suicide predictors in a general population and a clinical sample with machine learning

**DOI:** 10.1186/s12888-022-03702-y

**Published:** 2022-02-15

**Authors:** Lloyd D. Balbuena, Marilyn Baetz, Joseph Andrew Sexton, Douglas Harder, Cindy Xin Feng, Kerstina Boctor, Candace LaPointe, Elizabeth Letwiniuk, Arash Shamloo, Hemant Ishwaran, Ann John, Anne Lise Brantsæter

**Affiliations:** 1 Department of Psychiatry, University of Saskatchewan, Saskatoon, Canada; 2 College of Medicine, University of Saskatchewan, Saskatoon, Canada; 3 Diakonhjemmet Hospital, Oslo, Norway; 4 Mental Health & Addictions Services, Saskatchewan Health Authority, Saskatoon, Canada; 5 Department of Community Health and Epidemiology, Dalhousie University, Halifax, Canada; 6 Division of Biostatistics, University of Miami, Miami, USA; 7 Swansea University Medical School, Swansea University, Swansea, United Kingdom; 8 Department of Environmental Health, Norwegian Institute of Public Health, Oslo, Norway

**Keywords:** suicide, machine learning, prediction, primary prevention, secondary prevention

## Abstract

**Background:**

Machine learning (ML) is increasingly used to predict suicide deaths but their value for suicide prevention has not been established. Our first objective was to identify risk and protective factors in a general population. Our second objective was to identify factors indicating imminent suicide risk.

**Methods:**

We used survival and ML models to identify lifetime predictors using the Cohort of Norway (n=173,275) and hospital diagnoses in a Saskatoon clinical sample (n=12,614). The mean follow-up times were 17 years and 3 years for the Cohort of Norway and Saskatoon respectively. People in the clinical sample had a longitudinal record of hospital visits grouped in six-month intervals. We developed models in a training set and these models predicted survival probabilities in held-out test data.

**Results:**

In the general population, we found that a higher proportion of low-income residents in a county, mood symptoms, and daily smoking increased the risk of dying from suicide in both genders. In the clinical sample, the only predictors identified were male gender and older age.

**Conclusion:**

Suicide prevention probably requires individual actions with governmental incentives. The prediction of imminent suicide remains highly challenging, but machine learning can identify early prevention targets.

**Supplementary Information:**

The online version contains supplementary material available at (10.1186/s12888-022-03702-y).

## Background

In 2016, suicide was the second leading cause of death in the 15-29 age group and accounted for 793,000 deaths worldwide [1]. Suicide has a huge economic cost. In Spain, this was 6 billion euros annually (5 billion for men, 1 billion for women at 2013 prices) [2]. The cost for women is certainly underestimated because the value of housework and childcare is hard to estimate. Although men are increasingly involved in parenting, household duties are still largely shouldered by women [3].

Generally, suicide prevention programs focus on high-risk groups [4, 5] and high-risk periods [6]. There are studies about primary prevention programs in educational [7] or primary care settings but the quality of the evidence is hard to evaluate [8]. Suicide lags behind cardiovascular outcomes in primary prevention guidelines. Whereas healthy people know how to reduce their risk of cardiovascular disease overall, this is not true for suicide. For example, the American Heart Association (AHA) recommends 150 minutes of moderate physical activity (75 minutes of vigorous activity) per week for adults [9]. If people heed this advice, small reductions in blood pressure would translate into a lower incidence of coronary heart disease [10]. In effect, individuals become *agents of prevention* for cardiovascular events. By contrast, people receive no such guidance to reduce suicide risk. A UK study argued that broad population-based strategies result in greater suicide reductions than those focused on high-risk groups [11]. An example of a broad population approach is one hour of physical activity per week—this may prevent 12 percent of future depression cases [12]. Several modifiable suicide risks (discussed below) are already known and machine learning (ML) may identify additional ones.

Regarding secondary prevention, identifying high-risk patients is challenging. Clinicians cannot foresee which patients will act upon suicidal thoughts [13, 14]. Two reasons are: (1) suicidal thoughts do not progress linearly to suicide. [15] (2) Suicide-related outcomes (i.e. thoughts, attempts, and completions) have common risk factors [16]. As computers become cheaper and ubiquitous, ML is increasingly used for precision medicine, including the prediction of suicide [16–22]. ML can be defined as programs that learn from previous experience [23], in contrast to rule-based artificial intelligence that relies on programmer instructions [24].

There are known modifiable targets for suicide such as smoking [25–28], lipid and cholesterol profiles [29–31], dietary patterns [32], unemployment [33] and BMI in which overweight and obese individuals had lower suicide risk [34, 35]. Likewise, a meta-analysis reported that compared to those with normal weight, underweight people had higher suicide risk and overweight people had lower suicide risk [36]. We were unsure if these variables are causal or markers of suicidality. Nevertheless, each person has freedom of action subject to genetic, social, and environmental constraints [37]. Regarding the prediction of imminent suicide, the literature suggests that transitions in care are high risk periods [6]. These include: initial diagnosis with a mental condition, initiation of psychotropic medication, discharge from the hospital, and having a recent life-changing event [38, 39].

Previous ML papers used administrative data for suicide prediction [40, 41]. For example, Simon and colleagues examined about 3 million people who visited mental health and primary care centers to identify precursor events for suicidality [21]. An Australian group developed a risk score that accumulated information longitudinally and this score was shown to predict repeat episodes [42]. Both papers recommended using electronic health records to identify high-risk people. Whether it is worthwhile to do so is being debated. Belsher and colleagues systematically reviewed 17 studies and reported that the accuracy for predicting a future event is near zero [43]. However, this conclusion is disputed by Simon and colleagues, claiming that their model [21] has superior predictive value for imminent suicide compared to prediction models for breast cancer [44].

ML models could aid suicide prevention because these techniques combine the joint action of many risk factors without making typical statistical assumptions [45]. However, ML is not immune to other challenges in predicting suicide. Suicidal people may inadvertently or deliberately terminate their life [46] without presenting to care services. Also, the class imbalance problem—referring to data in which an outcome of interest is exceedingly rare compared to the other class is pervasive in suicide research [47]. Classifying every instance as a non-suicide would be correct most of the time but miss all the suicide cases whose deaths might otherwise have been prevented.

We had two main objectives in this study. First, we sought to identify early risk or protective factors for the primary prevention of suicide, especially those within each individual’s sphere of influence. Secondly, we examined if longitudinally collected records of mental health related hospital visits can predict suicides in a high-risk population.

## Materials and methods

The demographic characteristics of the participants of the general population and the clinical sample are presented in Table 1.

**Table 1:** Demographic characteristics of the study participants

	Cohort of Norway		Saskatoon	
N	173,224		13,892	
Sex				
Female	89,074	51%	7101	51%
Male	84,150	49%	6791	49%
Marital status				
Married / Partnered	104,665	60%	1413	10%
Single	38,470	22%	1120	8%
Divorced/separated/widowed	29,415	17%		
Missing	674	0%	11,359	82%
Suicide deaths	319	0%	80	1%
Mean age at entry (sd)	50	16	37	20
Mean follow-up time in years (sd)	16.68	4.71	3.11	1.19

Notes: For Saskatoon, the figures for Single are combined with Divorced/separated/widowed; Cell entries are frequencies (percentages) except for age and follow-up time.

### Ascertainment of suicide

The outcome variable in both the general population and clinical sample was suicide established by official authorities. For the Cohort of Norway, cause of death for deceased participants was provided to the research team as *suicide* or *other cause*. This was based on death certificates completed by a physician and entered into a national Cause of Death registry. Suicide is indicated by the ICD-10 codes X60-X84 and Y87.0 [48]. Of the 319 suicide deaths among cohort members, all were based on ICD-10 except for two deaths in 1995 that used ICD-9. From 2005 to 2014, three assessments regarding the quality of the data Norwegian Causes of Death Registry were made. The quality was classified in the second-best category (first two assessments) and in the best category (third assessment) [49].

For the Saskatoon data, the research team was provided with a list of suicide decedents (based on the same ICD-10 codes as Norway) by the provincial coroner. We are not aware of an external assessment of the mortality data from Saskatoon (and Canada in general) but the lack of a national standard and an accreditation system for coroner offices are notable weaknesses [50].

The research project was approved by University of Saskatchewan ethics board (Saskatoon data) and the Regionale Komiteer for Medisinsk og Helsefaglig Forskningsetikk (Norway data). All Cohort of Norway participants provided written consent to link their responses with government registers[51]. Consent to participate was waived for the Saskatoon data by the University of Saskatchewan (Approval number: Bio 17-11). Handling of both Norway and Saskatoon data adheres to the declaration of Helsinki.

### Cohort of Norway, population study

The Cohort of Norway (CONOR) study consisted of 11 health surveys carried out between 1994 and 2003 in various Norwegian regions [51]. CONOR included demographic data, self-reported medication use, lifestyle (diet and physical activity), smoking, alcohol consumption, and blood test results from 173,275 people who were between ages 18 to 105 at enrollment. For 7235 people who participated more than once, we used data from their initial participation only. Survey responses were linked with ICD-coded deaths up to December 31, 2016 by the Norwegian Institute of Public Health.

#### Candidate predictor variables

Our main candidate predictor for suicide was the sum of 7 questions regarding psychological health (mood symptoms). These were: felt nervous or worried, felt anxious, felt confident and calm, felt irritable, felt happy and optimistic, felt down, depressed,and felt lonely. These items are based on the Hopkins Symptom Checklist [52] which has been validated in various populations including Norway [53]. We reverse-coded the positively worded items before summation. The variables representing a healthy lifestyle and dietary factors were: engaging in light / hard physical activity, alcohol use (never or seldom, about monthly, more than monthly to once a week, several times a week), daily smoking, exposure to smoke-filled rooms, and exposure to secondary smoke as a child. We had the following biological measurements: triglycerides, HDL-cholesterol, glucose, total cholesterol (all in *μ*mol). The details regarding the collection of biomarkers and other characteristics are described in the cohort profile [51].

Other candidate predictors were: BMI, taking blood pressure medications, month of birth, having an injury requiring hospitalization, age, waist-hip-ratio, married status, and living with a spouse (partner). Although Norway is a welfare state, we included two measures of social status as predictors: years of education and relative social deprivation. A previous Norwegian study reported an association of higher psychological distress and low education [54]. Relative social deprivation was defined as the proportion of residents in a county with an after-tax income that is 50 percent below the median income or greater [55].

We likewise considered a wider range of suicide predictors but these had missing rates higher than 20 percent, beyond which imputation is not recommended [56]. These variables (missing rates) were: number of sleepless nights in a week (32%), having young children (79%), immigrant background (22%), number of good friends (24%), use of vitamins and supplements (66%), and taking antidepressants (70%).

### Saskatoon, Canada clinical sample

We created a retrospective cohort of people (n=12,614) who ever visited a Saskatoon hospital for a mental health or substance-related reason between 2011 and 2016. Using the first such visit as an index date, we constructed a longitudinal record of hospital and community visits for 4 years up to 31 March 2016 or until death by suicide, whichever came first. We required that people had at least 6 months of follow-up time, but included people dying of suicide in the first 6 months (n=13). We transformed this person-level data (1 row: 1 person) into a person-period dataset grouped into 6-month intervals. Each interval had time-varying predictors or suicide death (if applicable). These are explained in the next section.

#### Candidate predictor variables

Our main candidate predictor was the Repeated Episodes of Self-harm (RESH) score for each six-month interval [42]. RESH ranges from 0 to 25, with people scoring in the 20-25 range having over 80 percent risk of a repeat self-harm episode [42]. Although it was not developed for the purpose of suicide prediction, other studies have used the RESH components (psychiatric diagnoses [20, 21], hospitalizations [19], or self-harm episodes [40]) for suicide prediction. Aside from the RESH score, we had ICD diagnosis codes for each visit, intake and discharge dates, and whether the patient visited the emergency room only or was admitted as an inpatient. Each of 20 diagnosis fields was searched for the following ICD diagnoses: Substance misuse (F10-F19), Depression (F32-F39), Anxiety (F40-F49), Eating Disorder (F50), Personality disorders (F51-59), Schizophrenia and related (F20-F29), Mania (F30-F31), and ADHD (F90). We also include self-harm episodes not resulting in death, an indicator of high suicide risk [40], as a candidate predictor.

Just as with the CONOR, there were variables of interest to us but high missing proportions precluded their use. These variables (missing rates) were: highest educational attainment (63%), aboriginal status (70%), and area-level deprivation (28%).

## Analysis

Our analytical strategy can be summarized in seven steps:

1. Partitioning the data (CONOR or Saskatoon) into training and testing subsets. The training set was dedicated to developing survival and ML models while the testing set was held out to be later predicted by the trained models.
2. Balancing the training data such that equal numbers of suicides and non-suicides were represented. This would allow the statistical and ML models to detect predictors of suicide.
3. Imputing missing values in the training and test sets separately. Suicide status and time to death (or censorship) were not included in imputation.
4. Fitting univariate (multivariable) survival and ML models to the training data. The ML models were variations of random forests. These are described more fully in the Supplementary Material. For the Cohort of Norway, we developed separate models by gender because there were adequate numbers of suicide deaths, but not with Saskatoon data.
5. Identifying the top predictor variables.
6. Using the survival and ML models to predict survival probabilities in the test data.
7. Comparing the sensitivity, specificity, negative predictive value (NPV), positive predictive value (PPV), and area under the receiver operating characteristic (ROC) curve of the survival and ML models.

Readers who are interested in the technical details are referred to the Supplementary Material. There we present diagrams, tables of intermediate results, and the detailed accuracy measures (Step 7). We have also provided the R and Stata codes therein.

## Results

### Cohort of Norway

**Fig. 1:**
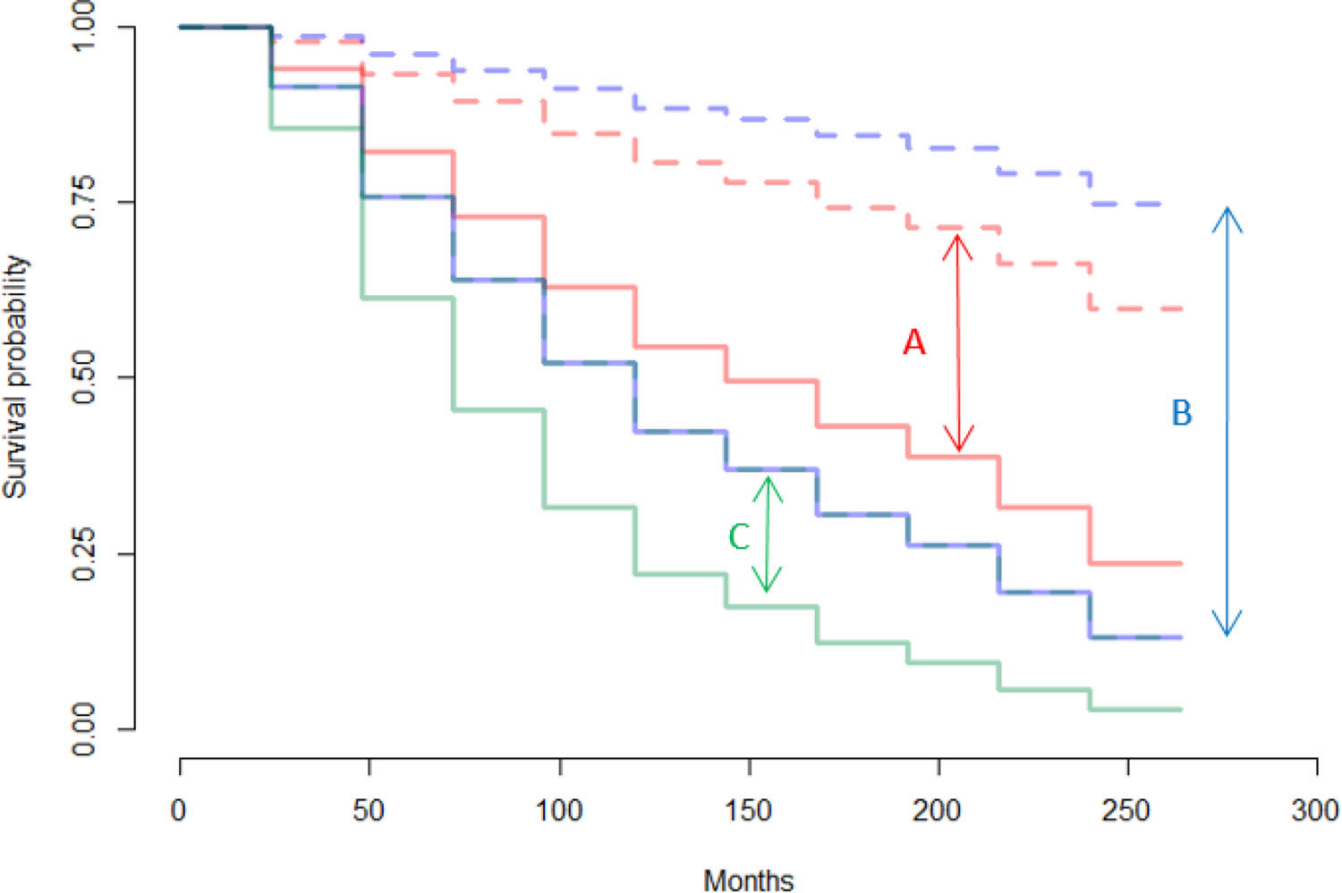
Predicted survival curves based on the Cox Model for Females (Cohort of Norway) **A:** Mood symptoms score: 7 (solid line) vs Mood symptoms score: 0 (dashed line) **B:** 8.3% low-income residents (solid line) vs. 3.0% low-income residents (dashed line) **C:** Daily smoker (solid line) vs Not daily smoker (dashed line, obscured by solid blue line)

**Fig. 2:**
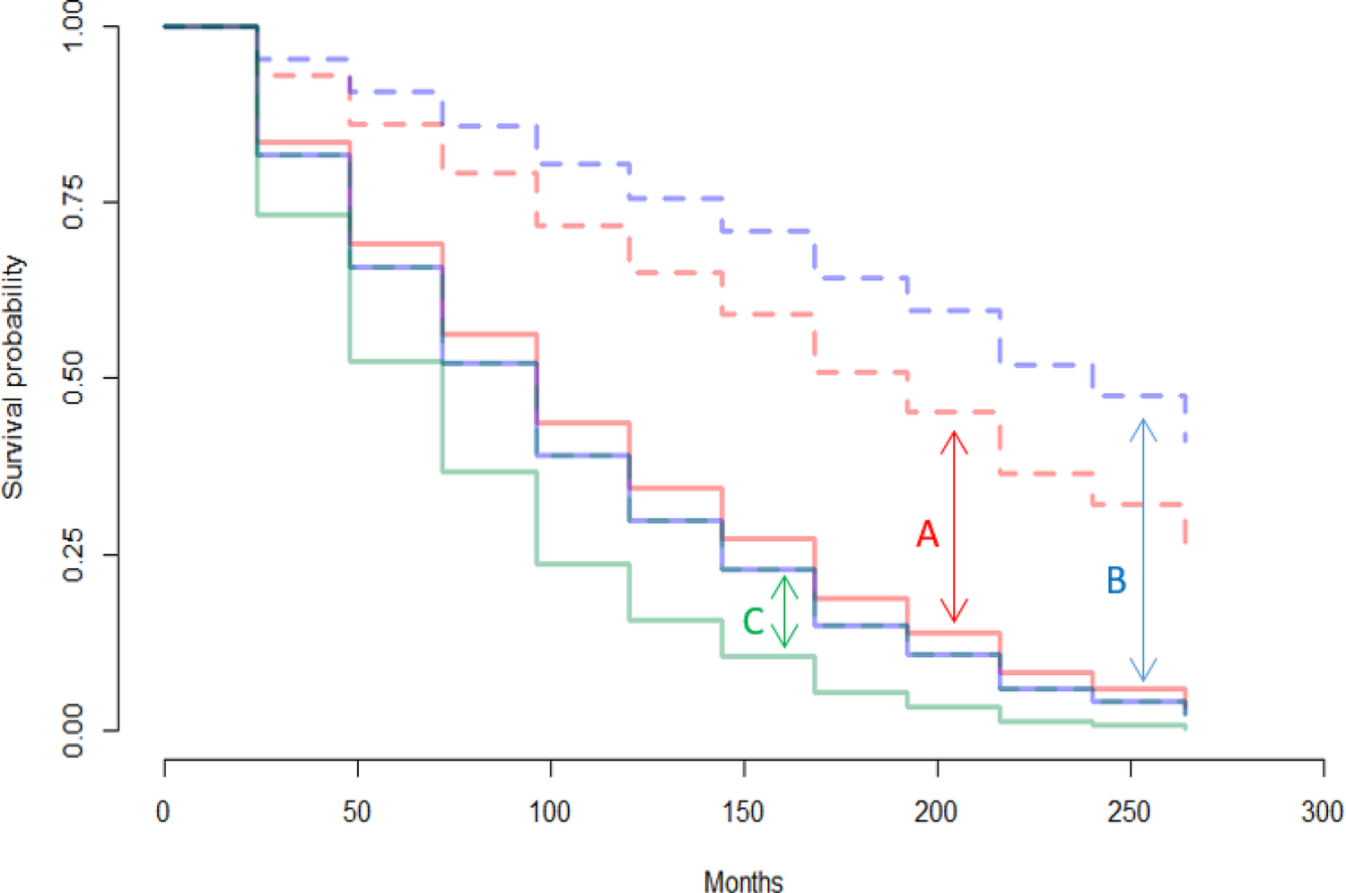
Predicted survival curves based on the Cox Model for Males (Cohort of Norway) **A:** Mood symptoms score: 7 (solid line) vs Mood symptoms score: 0 (dashed line) **B:** 8.3% low-income residents (solid line) vs. 3.0% low-income residents (dashed line) **C:** Daily smoker (solid line) vs Not daily smoker (dashed line, obscured by solid blue line)

In the Cohort of Norway, gender-separate Cox models showed that in women, higher age, higher proportion of low income residents, daily smoking, number of hours spent in smoke-filled rooms, and mood symptoms were associated with higher suicide risk. Being married was associated with lower suicide risk for women (Table 2). Among men, the risk factors were: higher proportion of low income residents, higher triglycerides, daily smoking, and mood symptoms (Table 3). A higher waist-hip ratio was associated with lower risk. The Cox model for females had an area under the ROC curve of 0.38 and for males it was 0.57 (Table S4 in the Supplement). Figure 1 shows the relative survival probabilities in females at high/low values of mood symptoms, low-income proportion, and daily smoking. Figure 2 shows the same comparison for males.

**Table 2:** Multivariable Cox Model for Females in the Cohort of Norway Training Data (*n*=235)

Variable	HR	95% CI	*p*
*Socio-demographic*			
Age	1.02	1.00-1.03	0.01
Married	0.64	0.44-0.94	0.02
Lives with spouse/partner	0.71	0.50-1.01	0.06
Proportion of county with low income	1.09	1.00-1.17	.04
*Physical/biological*			
Waist-hip ratio	0.98	0.66-14.70	0.99
Triglycerides	1.06	0.93-1.19	0.38
*Exposure to psychoactive substances and toxins*			
Daily smoking	1.52	1.08-2.14	0.02
Second hand smoke exposure in childhood	1.09	0.77-1.54	0.63
Daily hours spent in smoke-filled rooms	1.04	1.00-1.07	0.05
Alcohol use	1.02	0.88-1.18	0.81
*Physical activity and overall health*			
Frequency of hard physical activity at leisure time	1.09	0.94-1.25	0.24
Mood symptoms	1.11	1.02-1.21	0.01

**Table 3:** Multivariable Cox Model for Males in the Cohort of Norway Training Data (*n*=305)

Variable	HR	95% CI	*p*
*Socio-demographic*			
Age	1.01	0.99-1.02	0.48
Married	1.27	0.71-2.25	0.42
Lives with spouse/partner	0.61	.35-1.05	0.07
Proportion of county with low income	1.23	1.08-1.40	0.001
*Physical/biological*			
Waist-hip ratio	0.01	0.00-.66	0.03
Triglycerides	1.28	1.00-1.64	0.05
*Exposure to psychoactive substances and toxins*			
Daily smoking	2.06	1.21-3.51	0.02
Second hand smoke exposure in childhood	0.89	0.50-1.59	0.69
Daily hours spent in smoke-filled rooms	0.96	0.90-1.03	0.29
Alcohol use	1.22	0.97-1.53	0.09
*Physical activity and overall health*			
Frequency of hard physical activity at leisure time	0.98	0.78-1.23	0.83
Mood symptoms	1.18	1.05-1.32	0.02

The random survival forest for females identified the following significant predictors: higher proportion of low income residents, daily smoking, and mood symptoms (Table 4). The random survival forest model for males identified the same three variables and in addition, living with a spouse or a partner, being married, and taking blood pressure medications as protective factors (Table 4). The random survival forest model for females had area under the ROC curve of 0.50 and for males it was 0.43. (Table S4 in the Supplement)

**Table 4:** Top Predictors in the Random Survival Forest Model fitted to the Cohort of Norway Training Data

Combined Sexes (*n*=540)			Males Only (*n*=305)		Females Only (*n*=235)	
Variable	Importance	*p*	Importance	*p*	Importance	*p*
Male	1.45	<0.01	—	—	—	—
Proportion of county with low income	0.66	<0.01	0.50	0.03	0.63	0.01
Lives with spouse/partner	0.56	<0.01	0.58	0.01	—	—
Mood symptoms	0.56	<0.01	0.62	0.01	0.69	0.01
Daily hours spent in smoke-filled rooms	0.50	<0.01	0.57	0.03	—	—
Daily smoking	0.44	<0.01	0.36	0.01	0.56	<0.01
Waist-hip ratio	0.32	0.02	—	—	—	—
Married	0.27	0.01	0.71	<0.01	—	—
Alcohol use	0.20	0.05	—	—	—	—
Takes blood pressure medications	–	–	0.14	0.05	—	—

Note: Importance is determined by Altmann’s permutation method [57] in which the permuted values of a variable are compared with the true values. Greater decreases in prediction accuracy reflect higher importance.

### Saskatoon clinical sample

Of the univariate discrete survival models, only four models had interpretable odds ratios (ORs). These were the ones containing age, male sex, RESH score, and number of community mental health visits, each entered as a single predictor. Of these four models, higher age was the only factor that was associated with suicide death (Table 5). A one year increase in age at index increased suicide risk by 2 percent. The other variables had ORs that were infinitesimally small (e.g. for substance use, the OR was 1.97e-7).

**Table 5:** Univariate survival models in the Saskatoon Training data (*n* unique people = 134, *n* records = 777)

Variable	OR	95% CI	*p*
Age at index	1.02	1.00-1.03	0.01
Male	1.05	0.62-1.78	0.86
RESH score	0.95	0.71-1.27	0.72
Number of community mental health visits	1.16	0.88-1.51	0.29

*Note:* Each row is a single predictor in addition to interval (6-month periods, in days).

Since age alone had a *p* value <0.20 in univariate models, we did not create a multivariate model. We therefore used the univariate model to predict the held-out data for the probability of suicide at those intervals that contained at least 1 suicide death.

The historical random forest model identified only age at index and male gender as important predictors of suicide death (Table 6). The historical random forest model had an area under the ROC curve that was higher than that of logistic regression in 4 out of 5 intervals. However, both models had close to zero PPV in all intervals (Table S6 in the Supplement).

**Table 6:** Top Predictors in the Historical Random Forest model fitted to the Saskatoon Training data (*n* unique people = 134, *n* records = 777)

Predictor	Increase in Model Error if Predictor is marginalized	Z-score
Age at index	.009	-0.217
Male	.003	0.110

*Note:* Predictors are entered simultaneously in the model.

## Discussion

We fitted statistical and ML models to individual-level data in the Cohort of Norway and a clinical sample in Saskatoon, Canada. In the general population, we found that mood symptoms, daily smoking, and living in a county with a higher proportion of low income residents predict suicide death. These variables were consistently identified between sexes and by Cox and random survival forest models. In the clinical sample, no variables other than age and male gender predicted suicide at various follow-up intervals despite a longitudinal record of hospital visits.

### Long-term suicide prevention

The first implication of our general population result is that smoking abstinence or cessation is important for primary suicide prevention. It has been argued that the smoking-suicide association is spurious and that it can be explained by other causes such as substance abuse and mental disorders [58]. This seems to imply that smoking is a coping mechanism that is not of itself harmful to mood and cognition. An alternative explanation is that smoking is a psychological toxin that is not entirely accounted for by other suicide risks [59]. Several lines of evidence support this view. First is the dose-response relation between the quantity or intensity of smoking and suicide reported by large cohort studies [27, 60, 61]. Second is a Mendelian randomization study concluding that the associations of smoking, schizophrenia, and depression can partly be attributed to a causal effect of smoking [62]. Third, abstinence from smoking is associated with fewer suicide related outcomes, with a longer abstinence associated with lower suicide risk [63–65]. In a study that disentangled the genetic predisposition to smoke and smoking behaviour, a 35-year follow-up of twins in Finland reported that among twins, one of whom smoked and the other did not, death by suicide was more likely for the smoker [25]. Even though smoking is an individual choice, it is a public concern that the warning labels of cigarette boxes tend to focus on cancer risk, while remaining silent about mental health [62, 66]. Using a quasi-experimental approach, Grucza and colleagues [67] evaluated the impact of cigarette excise taxes and smoke-free air policies on suicide deaths. They concluded that an added $1 dollar excise tax on a pack of cigarettes translates to a 12.4% reduction in suicide risk. This shows that government policies are effective in nudging individuals into healthy behaviors.

The second implication is that mood symptoms should not be ignored, and seeking treatment is part of an individual’s duty of self-care. In both Norway and Canada, seeing a psychiatrist or psychologist is usually free. Unfortunately, there is no shortage of maladaptive beliefs preventing people from seeking help. Patients may fail to recognize their need for treatment or deny their illness [68, 69]. In the United States, where there is no universal health coverage, attitudinal barriers aggravate the limited access to health services [70]. Parents usually serve as gatekeepers to mental health services for their children [71], so receiving proper care often hinges on parental attitudes. Parents may refuse to seek care for their children for fear of the mental illness label [72]. They did so despite knowing that depression typically does not resolve on its own. People who have attempted suicide are more likely to seek help compared to counterparts who have a mental condition but no attempt [73]. A reason given for not seeking help is the desire to solve the problem by themselves [70, 73]. These behaviours hinder a timely provision of mental health care and ultimately increase the risk for suicide.

The finding that counties with higher proportion of low-income residents have higher suicide rates is consistent with a Norwegian case-control study that studied socio-economic predictors of suicide [74]. The study reported that suicide cases were overrepresented in people earning less than 400,000 NOK annually. Likewise, suicides were overrepresented in people with compulsory education only compared to tertiary education. These results are consistent with a Danish study showing that the lowest quartile of income was associated with higher suicide risk [75]. Low income and low socioeconomic status are known determinants of poor health outcomes [76]. Individuals with low socioeconomic status may have more unhealthy dietary patterns, smoke more, exercise less, are more often overweight and obese, resulting in poorer physical and mental health as a result. Further research is required to understand why these social determinants apply also to generous welfare regimes such as in the Scandinavian countries [77].

There were other variables that were protective for males only (higher waist-hip ratio) or females only (married). Overall, a higher waist-hip ratio is a risk for obesity, which two previous studies found to be associated with lower suicide risk [34, 35]. This finding needs to be further studied because obesity is an inflammatory condition and inflammation is associated with mood disorders [78]. Our results also identified risk factors for females only (hours in smoke-filled rooms) or males only (triglycerides). Both need to be studied further and it would be premature to interpret them at this time.

### Imminent suicide prevention

The main implication of our Saskatoon result is that predicting the timing of suicide is not feasible with hospital-based diagnosis alone and with small numbers of suicide cases. Note that our clinical sample was of reasonable size (N=13,892). However, this presumably high risk sample had too few suicide deaths (n=80) for ML to be effective. There were in fact 149 suicides in Saskatoon during the study period—a number that approximates Canada’s suicide incidence rate of 11.5 per 100,000 people per year [79]. The 69 other suicides never visited a Saskatoon hospital so we had no information about them. They may have had records from general practitioners, the police, social services, and forensic settings. Unfortunately, linking data across these settings is a formidable task in Canada because of inconsistent standards across provinces. Researchers have huge barriers to overcome before being granted access to research data, purportedly for privacy reasons [80]. It is possible that resources with: (1) a wider range of candidate variables, (2) coming from various agencies, (3) aggregated over longer periods, can enable the prediction of imminent suicide with greater accuracy. The SHRINE project in the USA is one such repository. SHRINE aggregates data about various diseases and makes electronic records available for research while preserving the privacy of patients [81].

### Accuracy of our prediction models

Our accuracy measures for the Cohort of Norway (Table S4) and Saskatoon (Table S6) were dismal overall. The areas under the ROC curve for the Cohort of Norway models were in the range: 0.38-0.57—roughly comparable to a random guess of a coin toss outcome. The positive predictive values (PPV) of our Cohort of Norway models were mostly 0, reaching a maximum of about 16 percent for male suicides (Cox model). Compared to the PPVs of 11 studies meta-analyzed by Belsher [43], ours was second best [82]. In contrast the AUCs of our Cohort of Norway models were below those of 7 studies that reported AUCs. The Saskatoon clinical sample models uniformly had PPVs close to zero. This is not surprising since there were no significant predictors aside from male sex and age, and few suicide cases. More surprising is that several studies [18, 19, 21, 83] with millions of patients also had PPVs close to zero. This does not imply that suicide prediction models are an exercise in futility. PPV depends on disease prevalence, and with suicide being rare, identifying true positives is extremely difficult. In effect, ML models may improve dramatically but their PPV is constrained ultimately by the problem of class imbalance.

### Limitations

The present study is subject to several limitations. First, we did not have data that included both self-reported health measures and healthcare utilization records. Having data that includes variables from both domains would help elucidate how primary and secondary preventive factors interact. Second, although we had a range of other variables in the Saskatoon clinical data, such as area-level deprivation, aboriginal status, highest level of education, and marital status, the missing rates were unacceptably high, so we decided not to use them as predictors. Third, we deviated from the usual practice in suicide studies to combine suicide deaths with those that the coroner ruled as “undetermined intent” so our outcome variable excludes suicides that the coroner could not ascertain. Fourth, non-fatal self-harm episodes (X60-X84) do not distinguish between events with and without an intent to die [21]. This means that emergency room visitors who cut themselves as a form of coping are assigned the same ICD code as visitors who hanged themselves but survived. This most likely diluted the predictive value of self-harm for future suicides. Finally, there may have been some leakage of information from the test to the training set during imputation. This may have resulted in a slight inflation of prediction accuracy.

### Conclusion

Suicide prevention probably requires individual actions with governmental incentives. The prediction of imminent suicide remains highly challenging, but machine learning can identify early prevention targets.

## Supplementary Information

**Additional file 1** Analytical details for the cohort of Norway. Analytical details for the Saskatoon clinical sample. R code

## Data Availability

The data that support the findings of this study are available from the Norwegian Institute of Public Health and the Saskatchewan Health Authority but restrictions apply to the availability of these data, which were used under license for the current study, and so are not publicly available. With the permission of the Norwegian Institute of Public Health and the Saskatchewan Health Authority, the corresponding author will make the data available upon reasonable request.
