# Supplementary material for: Identifying long-term and imminent suicide predictors in a general population and a clinical sample with machine learning
Source: BMC Psychiatry. 2022 Feb 15;22:120. doi: 10.1186/s12888-022-03702-y (PMC8848909; doi:10.1186/s12888-022-03702-y)
Supplement: Supplementary file 1 — Additional file 1 Analytical details for the cohort of Norway. Analytical details for the Saskatoon clinical sample. R code [file 12888_2022_3702_MOESM1_ESM.pdf]

# Supplement to *Identifying long-term and imminent suicide predictors in a general population and a clinical sample with machine learning*

Lloyd Balbuena, Marilyn Baetz, Joseph Andrew Sexton, Douglas Harder, Cindy Xin Feng, Kerstina Boctor, Candace LaPointe, Elizabeth Letwiniuk, Arash Shamloo, Hemant Ishwaran, Ann John & Anne Lise Brantsæter

## 1. ANALYTICAL DETAILS IN THE COHORT OF NORWAY

### Steps 1 and 2: Partitioning and Balancing

We first partitioned the Cohort of Norway data into training and testing subsets, assigning 85 percent of the suicide cases to training (n=270) and 15 percent to testing (n=49). Imbalanced data—where one class predominates over the other makes it difficult for machine learning algorithms to predict the minority outcome [1]. Accordingly, we randomly sampled 270 individuals who did not die from suicide (i.e. alive or died from other causes) and added them to the training set, thereby giving us a balanced set of suicides and non-suicides. The testing subset consisted of the rest of the suicide cases (n=49) plus the rest of the Cohort of Norway participants. Partitioning the data in this manner resulted in a small training set (n=540) and a large test set (n=172,684). In the sex-segregated models, the split in the training set was 305 males/235 females and 83,845 males/88,839 females in the testing set.

### Steps 3: Imputation

We examined the missing data patterns, and discarded nine variables with a missing proportion exceeding 20 percent. According to Liao and colleagues [2], 20 percent is typical practice and researchers should not be overconfident that highly missing variables are imputable. The discarded variables (and their missing proportions) were: living with others with ages 18 and below (52%), having children of kindergarten age (78%), using vitamin/mineral supplements (69%), takes lipid lowering drugs (65%), number of good friends (24%), number of months taking antidepressant in the previous year (70%), lack of sleep has affected work (33%), frequency of attendance in organized activity (28%), and frequency of sleeplessness (32%). The remaining variables had missing rates below 21 percent (or no missing values at all) except for having an injury requiring hospitalization (21%) which was close to the threshold. This variable was retained, in part, because some of them may have been self-harm injuries and potentially informative about suicide.

We adhered to two principles in carrying out imputation: (1) preventing data leakage between training and testing sets, and (2) preventing the outcome (suicide death, and survival time) from influencing the imputation of predictors. Since our primary objective was to identify predictors of suicide, using suicide status and follow-up time to impute missing predictors would be circular. Accordingly, we imputed training and test sets separately and excluded the outcome variables from imputation models. Having continuous, ordinal, and dichotomous variables, we chose two methods shown to be appropriate for datasets with mixed variable types. These are missForest [3] and Factorial Analysis for Mixed data (FAMD) [4], an adaptation of principal components analysis to mixed data types. We had a bake-off between FAMD and missForest, comparing the accuracy of each method for each candidate variable, at five different missing proportions: 5, 10, 15, and 20 percent. We then used the method having greater overall accuracy to impute a particular variable. This follows the strategy of Liao and colleagues [2] that selects a particular method that performs better (i.e. higher accuracy) for a particular variable.

The assessment of accuracy was determined in the following manner. Two identical copies of the testing dataset were created–one for imputation using missForest and the other using FAMD. We used the testing data for assessment of accuracy (instead of the training data) because it was

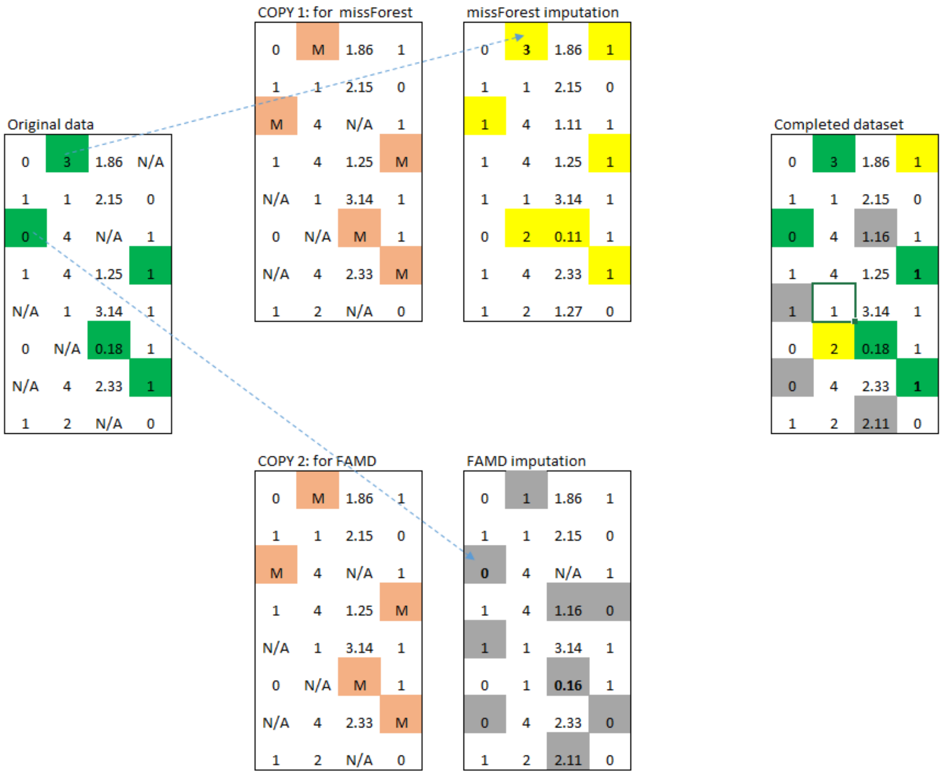


**Fig. S1.** Two identical copies of the original data (left) were created and allocated for imputation by missForest and FAMD (middle). Since original data also has missing values, assessment of accuracy is restricted to values that were not missing in the original. We masked them (pink highlight) to serve as ground truth. Accuracy is defined by the imputed value (gray or yellow highlight) that has a smaller error with respect to the true values (green highlight). Dashed arrows point to examples of imputed values with greater accuracy. The method with greater accuracy is then used to impute the missing values in the original data, resulting in a completed dataset.Highlights in the completed data (right) indicate the source of a particular figure, i.e. missForest (yellow), FAMD (pink), or original data (green).

large, and enabled us to mask (or hide) known data. Accuracy was assessed by comparing values of the masked data with the original data, using appropriate metrics: RMSE for continuous and ordinal variables [5] and AUC for dichotomous variables. Figure S1 is a schematic diagram of the masking procedure and accuracy assessment procedure.

We found that imputation accuracy remained stable at various missing proportions in the 5 to 20 percent range (Table S1). Generally, missForest had greater accuracy for dichotomous variables and FAMD for ordinal and continuous ones. Once we found which method worked better for a particular variable in the testing subset, we used that same imputation method in the training subset.

### Step 4: Fitting survival and ML models to the training data

We first developed Cox models to the training data, fitting each of 13 predictors in a univariate model. Predictors that were significant at $p = .2$ were entered into multivariable Cox models—-one for males and one for females, consistent with previous work [6, 7]. We tested the proportional hazards assumption in these Cox models and found no violation.

We then fitted random survival forests to the same training data using the `randomForestSRC` package [8]. The predictive ability of random forests depends on so-called hyper-parameters (i.e. number of variables to try, number of observations per terminal node, number of split points per variable) specified by the user. Accordingly, we selected the optimal hyper-parameters in a

**Table S1.** Imputation Accuracy in Dichotomous Variables

| Variable | Proportion Missing (%) in the testing data | Masked Proportion (%) | FAMD | missForest | Winning method |
|---|---|---|---|---|---|
| | | | Area under ROC curve | | |
| 1. Male | 0 | N/A | N/A | N/A | No missing values, not imputed |
| 2. Injury requiring hospitalization | 20.81 | 5 | 0.5 | **0.5** | missForest, due to lower S.E. |
| | | 10 | 0.5 | **0.5** | |
| | | 15 | 0.5 | **0.5** | |
| | | 20 | 0.5 | **0.5** | |
| 3. Smokes daily | 10.08 | 5 | 0.64 | **0.73** | missForest |
| | | 10 | 0.64 | **0.73** | |
| | | 15 | 0.63 | **0.72** | |
| | | 20 | **0.63** | 0.6 | |
| 4. Lives with spouse or partner | 0 | N/A | N/A | N/A | No missing values, not imputed |
| 5. Married | 0.39 | 5 | 0.5 | **0.71** | missForest |
| | | 10 | 0.5 | **0.68** | |
| | | 15 | 0.5 | **0.67** | |
| | | 20 | 0.5 | **0.65** | |
| 6. Exposed to second-hand smoke in childhood | 16.78 | 5 | **0.53** | 0.51 | FAMD |
| | | 10 | **0.54** | 0.53 | |
| | | 15 | **0.54** | 0.51 | |
| | | 20 | **0.54** | 0.51 | |
| 7. Takes blood pressure medications | 1.02 | 5 | **0.51** | 0.5 | FAMD |
| | | 10 | **0.51** | 0.5 | |
| | | 15 | 0.5 | **0.51** | |
| | | 20 | **0.51** | 0.5 | |

**Table S2.** Imputation Accuracy in Ordinal Variables

| Variable | Proportion Missing (%) in the testing data | Masked Proportion (%) | FAMD | missForest | Winning method |
|---|---|---|---|---|---|
| | | | Root MSE | | |
| 1. Alcohol use (Never/seldom, 1xmonth, 2-3xmonth-1xweek, >1xweek) | 9.96 | 5 | **1.03** | 1.04 | FAMD |
| | | 10 | **1.03** | 1.06 | |
| | | 15 | **1.03** | 1.04 | |
| | | 20 | **1.03** | 1.06 | |
| 2. Hours of light physical activity at leisure per week (None, <1hr, 1-2hrs, 3+hrs) | 9.35 | 5 | **0.93** | 0.95 | FAMD |
| | | 10 | **0.93** | 0.95 | |
| | | 15 | **0.93** | 0.95 | |
| | | 20 | **0.92** | 0.94 | |
| 3. Hours of hard physical activity at leisure per week (None, <1hr, 1-2hrs, 3+hrs) | 15.57 | 5 | **1.02** | 1.05 | FAMD |
| | | 10 | **1.02** | 1.04 | |
| | | 15 | **1.02** | 1.04 | |
| | | 20 | **1.03** | 1.06 | |

tuning process that resulted in the smallest out-of-bag error.

### Step 5: Top Predictor Variables

We examined the important predictor variables, separately by gender, in both Cox and random survival forest models. In the Cox models, we focused on variables that were statistically significant at p = .05. For the random survival forest, we used Altmann's permutation method, [9] selecting a *p* value threshold of .05. With this method, the importance of a variable is based on the decrease in prediction accuracy when the outcome is permuted vis-a-vis the true values [9]. This method is resilient to the influence of variables with many categories. We implemented Altmann's permutation method using the `ranger` package [10].

### Step 6-7: Predicting the Test Set Outcomes and Assessment of Accuracy

Using the models developed in Step 4, we predicted the survival probabilities of the observations in the test set at the first, second, third and fourth quartiles. These values were: 181, 212, 243, and 267 months. The models' predictive accuracy were assessed using Heagerty's cumulative cases /dynamic controls method [11]. This method is intended for the assessment of predictors that were measured at participants' entry. Sensitivity, specificity, positive predictive value (PPV), and negative predictive value (NPV) at each quartile were calculated based on an optimal threshold, selected using the criterion proposed by Perkins and Schisterman [12]. This threshold represents the value of the marker that results in the point closest to the upper left corner of the ROC curve (i.e. (1,0)). We also calculated the area under the ROC curve (i.e. the C-index) for the last follow-up period. The calculation of accuracy measures was performed in Stata using the package `stroccurve` [13]. The accuracy measures for the Cohort of Norway test set are in Table S4.

## 2. SASKATOON CLINICAL SAMPLE

### Steps 1 and 2: Partitioning and Balancing

The format of the person-period data for Saskatoon is shown in Table S5. This individual has four rows corresponding to the six-month intervals (in days) in which the person visited the hospital. Along with the fixed variables *Age* and *Male*, the mental health conditions associated with each visit are recorded. `Person 1` was seen for anxiety in the first six months and for depression in the three succeeding interval. The person died of suicide sometime between the third and fourth 6-month interval.

As we did in the Cohort of Norway analysis, the Saskatoon data was partitioned into training and testing subsets. The training data consisted of 67 suicide deaths and 67 living people—giving 134 unique people having a mean follow-up time of 2.5 years (SD: 1.4). Because of multiple records per person, the training data had 777 rows. The testing data had 13 suicide deaths and 12,473 controls with a mean follow-up time of 3.1 years (SD: 1.2). The testing data had 72,991 rows because of multiple records per person.

### Step 3: Imputation

In Saskatoon, all hospital visits are captured electronically, so there were no missing values with respect to the diagnoses and the RESH score. The person-level characteristics had high rates of missingness, so these were not imputed.

### Step 4: Fitting survival and ML models to the training data

We fit two types of models to the Saskatoon training data: a discrete time survival model, and a historical random forest, which uses historical and current interval predictors. The historical random forest is similar to a survival model with time-varying predictors, but uses these variables to "grow" decision trees for predicting suicide risk. The historical random forest was implemented in the `htree` package [14].

The discrete survival model was implemented as logistic regression with the intervals treated as factors. We entered fixed and time-varying covariates one at a time to the model with only intervals as predictors. Variables that were significant at p = .20 were entered into a multivariate logistic model. For the historical random forest, we entered the following as historical predictors: self-harm, anxiety, depression, substance abuse, schizophrenia, mania, ADHD episodes, RESH score, and previous community mental health visits. For concurrent predictors, we entered only gender and age at index. We set the terminal node size at 13 (i.e. 10 percent of the training sample) based on the recommendation by Dankowski [15].

**Table S3.** Imputation Accuracy in Continuous Variables

| Variable | Proportion Missing (%) in the testing data | Masked Proportion (%) | FAMD | missForest | Winning method |
|---|---|---|---|---|---|
| | | | Root MSE | | |
| 1. Month of birth | 0 | 5 | N/A | N/A | No missing values |
| | | 10 | | | |
| | | 15 | | | |
| | | 20 | | | |
| 2. Age at interview | 0 | 5 | N/A | N/A | No missing values |
| | | 10 | | | |
| | | 15 | | | |
| | | 20 | | | |
| 3. Waist-hip ratio | 12.49 | 5 | 5 | 0.061 | missForest |
| | | 10 | 10 | 0.062 | |
| | | 15 | 15 | 0.063 | |
| | | 20 | 20 | 0.064 | |
| 4. Proportion of county with low income | 0.27 | 5 | 5 | 1.83 | FAMD |
| | | 10 | 10 | 1.87 | |
| | | 15 | 15 | 1.88 | |
| | | 20 | 20 | **1.86** | |
| 5. Hours spent daily in smoke-filled rooms | 8.8 | 5 | **3.24** | 3.33 | FAMD |
| | | 10 | **3.4** | 3.53 | |
| | | 15 | **3.4** | 3.46 | |
| | | 20 | **3.38** | 3.45 | |
| 6. Count of mood symptoms (0-7) | 0 | 5 | N/A | N/A | No missing values |
| | | 10 | | | |
| | | 15 | | | |
| | | 20 | | | |
| 7. Triglycerides | 0.22 | 5 | 0.96 | **0.94** | FAMD |
| | | 10 | **0.99** | 1.07 | |
| | | 15 | **1.06** | 1.07 | |
| | | 20 | 1 | 1 | |
| 8. Glucose | 11.53 | 5 | **1.49** | 1.53 | FAMD |
| | | 10 | **1.35** | 1.39 | |
| | | 15 | **1.48** | 1.51 | |
| | | 20 | **1.43** | 1.46 | |
| 9. HDL-cholesterol | 0.22 | 5 | 0.36 | 0.36 | missForest |
| | | 10 | 0.36 | 0.36 | |
| | | 15 | 0.36 | **0.34** | |
| | | 20 | 0.36 | **0.34** | |
| 10. Total cholesterol | 0.18 | 5 | 1.12 | **1.11** | missForest |
| | | 10 | **1.14** | 1.15 | |
| | | 15 | 1.18 | **1.15** | |
| | | 20 | 1.14 | **1.12** | |
| 11.BMI | 0.63 | 5 | **3.67** | 3.71 | FAMD |
| | | 10 | **3.7** | 3.86 | |
| | | 15 | **3.74** | 3.75 | |
| | | 20 | 3.73 | **3.69** | |
| 12.Years smoked as a fraction of age | 0 | 5 | N/A | N/A | No missing values |
| | | 10 | | | |
| | | 15 | | | |
| | | 20 | | | |
| 13.Years of education (0-88) | 3.5 | 5 | **3.1** | 3.16 | FAMD |
| | | 10 | **3.13** | 3.26 | |
| | | 15 | **3.08** | 3.13 | |
| | | 20 | **3.13** | 3.21 | |

**Table S4.** Accuracy Measures for the Cohort of Norway Test Set

| | | | Males, Cox Model | | | | |
|---|---|---|---|---|---|---|---|
| Time | Cases | At risk | Se (%) | Sp (%) | PPV (%) | NPV (%) | AUC* |
| Qtl 1: 0- 181 months | 30 | 60737 | 0.574 | 0.689 | 0.001 | 1.000 | 0.57 |
| Qtl 2: 181-212 months | 33 | 39652 | 0.553 | 0.684 | 0.001 | 0.999 | |
| Qtl 3: 212-243 months | 34 | 19772 | 0.524 | 0.684 | 0.003 | 0.999 | |
| Qtl 4: 243-267 | 35 | 447 | 0.695 | 0.684 | 0.157 | 0.964 | |
| | | | | | | | |
| | | | Males, Random Survival Forest | | | | |
| Time | Cases | At risk | Se (%) | Sp (%) | PPV (%) | NPV (%) | AUC* |
| Qtl 1: 0- 181 months | 30 | 60737 | 0.593 | 0.666 | 0.001 | 1.000 | 0.43 |
| Qtl 2: 181-212 months | 33 | 39652 | 0.657 | 0.567 | 0.001 | 0.999 | |
| Qtl 3: 212-243 months | 34 | 19772 | 0.622 | 0.567 | 0.002 | 0.999 | |
| Qtl 4: 243-267 | 35 | 447 | 0.781 | 0.483 | 0.114 | 0.963 | |
| | | | | | | | |
| | | | Females, Cox Model | | | | |
| Time | Cases | At risk | Se (%) | Sp (%) | PPV (%) | NPV (%) | AUC* |
| Qtl 1: 0- 181 months | 11 | 68660 | 0.755 | 0.425 | 0.000 | 1.000 | 0.38 |
| Qtl 2: 181-212 months | 14 | 46248 | 0.804 | 0.425 | 0.000 | 1.000 | |
| Qtl 3: 212-243 months | 14 | 22287 | 0.804 | 0.425 | 0.001 | 1.000 | |
| Qtl 4: 243-267 months | 14 | 439 | 0.804 | 0.425 | 0.044 | 0.985 | |
| | | | | | | | |
| | | | Females, Random Survival Forest | | | | |
| Time | Cases | At risk | Se (%) | Sp (%) | PPV (%) | NPV (%) | AUC* |
| Qtl 1: 0- 181 months | 11 | 68660 | 0.831 | 0.564 | 0.000 | 1.000 | 0.50 |
| Qtl 2: 181-212 months | 14 | 46248 | 0.782 | 0.564 | 0.001 | 1.000 | |
| Qtl 3: 212-243 months | 14 | 22287 | 0.782 | 0.564 | 0.001 | 1.000 | |
| Qtl 4: 243-267 months | 14 | 439 | 0.782 | 0.564 | 0.056 | 0.987 | |

*Notes:* *AUCs are calculated at the last follow-up (267 months).

**Table S5.** Format of Saskatoon clinical data

| Person ID | Days | Suicide | Age at Index | Male | Depression | Anxiety | . . . |
|---|---|---|---|---|---|---|---|
| 1 | 183 | 0 | 25 | 1 | 0 | 1 | |
| 1 | 366 | 0 | 25 | 1 | 1 | 0 | |
| 1 | 549 | 0 | 25 | 1 | 1 | 0 | |
| 1 | 732 | 1 | 25 | 1 | 1 | 0 | |

**Table S6.** Accuracy Measures for the Saskatoon Clinical Sample Test Set

| | | | Discrete Survival Model, (Combined Sexes) | | | | |
|---|---|---|---|---|---|---|---|
| Time* | Cases | At risk | Se (%) | Sp (%) | PPV (%) | NPV (%) | AUC |
| Interval: 0-183 | 3 | 12,486 | 0.616 | 0.257 | 0.000 | 1.000 | 0.542 |
| Inteval: 183-366 | 1 | 12,483 | 0.735 | 0.257 | 0.000 | 1.000 | 0.545 |
| Interval: 366-549 | 3 | 11,212 | 0.864 | 0.257 | 0.000 | 1.000 | 0.68 |
| Interval: 732-915 | 3 | 8,301 | 0.515 | 0.536 | 0.000 | 1.000 | 0.566 |
| Interval: 915-1098 | 3 | 6,885 | 0.562 | 0.655 | 0.001 | 1.000 | 0.569 |
| | | | | | | | |
| | | | Historical Random Forest (Combined Sexes) | | | | |
| Time* | Cases | At risk | Se (%) | Sp (%) | PPV (%) | NPV (%) | AUC |
| Interval: 0-183 | 3 | 12,486 | 0.677 | 0.899 | 0.002 | 1.000 | 0.569 |
| Inteval: 183-366 | 1 | 12,483 | 0.768 | 0.713 | 0.000 | 1.000 | 0.644 |
| Interval: 366-549 | 3 | 11,212 | 0.704 | 0.713 | 0.001 | 1.000 | 0.697 |
| Interval: 732-915 | 3 | 8,301 | 0.685 | 0.657 | 0.001 | 1.000 | 0.585 |
| Interval: 915-1098 | 3 | 6,885 | 0.841 | 0.471 | 0.001 | 1.000 | 0.532 |

** Times are restricted to intervals with at least one suicide*

**Step 5: Top Predictor Variables**

For the discrete survival model, only age was as a significant predictor, so it was not necessary to create a multivariable model. For the historical random forest method, importance was determined by integrating predictors one at a time. The prediction errors of the original model and the marginalized model were then compared and the *p* value and z-score of the difference was calculated. This procedure implemented using the variable importance function in `htree`.

**Steps 6-7: Predicting the Test Set Outcomes and Assessment of Accuracy**

Using the models developed in Step 4, the survival probabilities for each person in the test set was predicted at quartiles of follow-up time. We restricted the assessment of accuracy to those intervals where at least one suicide death occurred. This was done to avoid zero PPV values resulting from zero positive cases. In contrast to the Cohort of Noway, we followed the incident cases/dynamic controls method because our Saskatoon data had updated measurements at each interval [16]. Sensitivity, specificity, PPV, NPV at each of the intervals with suicide were also calculated using `stroccurve`. Unlike the Cohort of Norway analysis, we did not create separate models by sex because we were limited by the number of suicide deaths. The accuracy measures for the Saskatoon clinical sample are summarized in Table S6.

## 3. R CODE FOR IMPLEMENTING THE ANALYSIS

### Cohort of Norway

***Steps I & II: Partitioning and balancing***

```
library(caret)
set.seed(66)
tr_index <-createDataPartition(conor_anon$suicide, p=0.85, list=FALSE, times=1)

conor_small <- conor_anon[tr_index,]
conor_large <- conor_anon[-tr_index,]

#the training file needs to contain equal numbers of suicides/non-suicides
```

***Step III: Imputation***

```
#this groups the CONOR variables into different types in order to
easily use the same accuracy metric for each type
library(tidyverse)
#define the variable types and do this for both test (conor_large)
and training (conor_small) sets.
```

```
ord_fac <- conor_large %>% select(alc_use,
                                   freq_hard_act_leis,
                                  freq_light_act_leis)

contin <- conor_large %>% select(age,waist_hip_ratio, low_income_pct,
                                hrs_in_smoke_rooms, mood_symps,
                                trig_mmol, gluc_mmol, hdl_mmol,
                                tot_chol_mmol, yrs_smk_frac_life,
                                yrs_educ, bmi, month_born)


dichot <- conor_large %>% select(injur_hosp, smoke_daily,
                                male, live_w_spouse,
                                married, sec_smoke_chld,
                                bp_med)

#Repeat the above code for conor_small
#Induce artificial missing values (i.e. mask them and impute for later
comparison with true value). Do this at 5, 10, 15, and 20 percent missing.

###FAMD Imputation###
library(doParallel)
cores <- detectCores()

library(missForest)
library(missMDA)
library(missRanger)
library(mvtnorm)

##################20 pct
conor_l_anon <- conor_large[,-c(1:3)]
conor_l_miss_20 <- prodNA(conor_l_anon, noNA=.20)

conor_imp20_FAMD <-imputeFAMD(conor_l_miss_20, ncp=3)
conor_imp20_FAMD <-conor_imp20_FAMD$completeObs

#####################15 pct
conor_l_miss_15 <- prodNA(conor_l_anon, noNA=.15)

conor_imp15_FAMD <-imputeFAMD(conor_l_miss_15, ncp=3)
conor_imp15_FAMD <-conor_imp15_FAMD$completeObs

#####################10 pct
conor_l_miss_10 <- prodNA(conor_l_anon, noNA=.10)

conor_imp10_FAMD <-imputeFAMD(conor_l_miss_10, ncp=3)
conor_imp10_FAMD <-conor_imp10_FAMD$completeObs

#####################5 pct
conor_l_miss_05 <- prodNA(conor_l_anon, noNA=.05)

conor_imp05_FAMD <-imputeFAMD(conor_l_miss_05, ncp=3)
conor_imp05_FAMD <-conor_imp05_FAMD$completeObs


###MissForest Imputation###

conor_imp20_MR <- missRanger(conor_l_miss_20, pmm.k = 3, num.trees=1000,
                            splitrule = "extratrees", seed = -13, verbose=2,
```

```
                            sample.fraction=.2)
conor_imp15_MR <- missRanger(conor_l_miss_15, pmm.k = 3, num.trees=1000,
                            splitrule = "extratrees", seed = -13, verbose=2,
                            sample.fraction=.2)
conor_imp10_MR <- missRanger(conor_l_miss_10, pmm.k = 3, num.trees=1000,
                            splitrule = "extratrees", seed = -13, verbose=2,
                            sample.fraction=.2)
conor_imp05_MR <- missRanger(conor_l_miss_05, pmm.k = 3, num.trees=1000,
                            splitrule = "extratrees", seed = -13, verbose=2,
                            sample.fraction=.2)

#After comparing the imputation accuracy of FAMD and missForest
for each level of missingness, replace the actual NA values with the imputed ones
at 5 percent missing rate.

#For these variables, missForest was more accurate

conor_l_comp <- conor_large
conor_l_comp$injur_hosp <- ifelse(is.na(conor_l_comp$injur_hosp),
                                conor_imp05_MR$injur_hosp,
                                conor_l_comp$injur_hosp)

conor_l_comp$smoke_daily <- ifelse(is.na(conor_l_comp$smoke_daily),
                                conor_imp05_MR$smoke_daily,
                                conor_l_comp$smoke_daily)

conor_l_comp$married <- ifelse(is.na(conor_l_comp$married),
                               conor_imp05_MR$married, conor_l_comp$married)

conor_l_comp$hdl_mmol <- ifelse(is.na(conor_l_comp$hdl_mmol),
                                conor_imp05_MR$hdl_mmol,
                                conor_l_comp$hdl_mmol)

conor_l_comp$tot_chol_mmol <- ifelse(is.na(conor_l_comp$tot_chol_mmol),
                                    conor_imp05_MR$tot_chol_mmol,
                                    conor_l_comp$tot_chol_mmol)

#For these variables, FAMD was more accurate

conor_l_comp$sec_smoke_chld <- ifelse(is.na(conor_l_comp$sec_smoke_chld),
                                    conor_imp05_FAMD$sec_smoke_chld,
                                    conor_l_comp$sec_smoke_chld)

conor_l_comp$sec_smoke_chld <-factor(conor_l_comp$sec_smoke_chld,
                                    labels = c("No", "Yes"))

conor_l_comp$bp_med <- ifelse(is.na(conor_l_comp$bp_med),
                              conor_imp05_FAMD$bp_med, conor_l_comp$bp_med)

conor_l_comp$bp_med<-factor(conor_l_comp$bp_med, labels = c("No", "Yes"))

conor_l_comp$alc_use <- ifelse(is.na(conor_l_comp$alc_use),
                             conor_imp05_FAMD$alc_use,
                             conor_l_comp$alc_use)

conor_l_comp$alc_use <- ordered(conor_l_comp$alc_use)

conor_l_comp$freq_light_act_leis <-ifelse(is.na(conor_l_comp$freq_light_act_leis),
                                        conor_imp05_FAMD$freq_light_act_leis,
```

```
                                        conor_l_comp$freq_light_act_leis)

conor_l_comp$freq_light_act_leis <- ordered(conor_l_comp$freq_light_act_leis)

conor_l_comp$freq_hard_act_leis <-ifelse(is.na(conor_l_comp$freq_hard_act_leis),
                                        conor_imp05_FAMD$freq_hard_act_leis,
                                        conor_l_comp$freq_hard_act_leis)

conor_l_comp$freq_hard_act_leis <- ordered(conor_l_comp$freq_hard_act_leis)

conor_l_comp$low_income_pct <- ifelse(is.na(conor_l_comp$low_income_pct),
                                    conor_imp05_FAMD$low_income_pct,
                                    conor_l_comp$low_income_pct)

conor_l_comp$hrs_smoke_rooms <- ifelse(is.na(conor_l_comp$hrs_smoke_rooms),
                                    conor_imp05_FAMD$hrs_smoke_rooms,
                                    conor_l_comp$hrs_smoke_rooms)

conor_l_comp$trig_mmol <- ifelse(is.na(conor_l_comp$trig_mmol),
                                 conor_imp05_FAMD$trig_mmol,
                                 conor_l_comp$trig_mmol)

conor_l_comp$gluc_mmol <- ifelse(is.na(conor_l_comp$gluc_mmol),
                                 conor_imp05_FAMD$gluc_mmol,
                                 conor_l_comp$gluc_mmol)

conor_l_comp$yrs_educ <- ifelse(is.na(conor_l_comp$yrs_educ),
                                conor_imp05_FAMD$yrs_educ,
                                conor_l_comp$yrs_educ)

conor_l_comp$bmi <- ifelse(is.na(conor_l_comp$bmi),
                           conor_imp05_FAMD$bmi,
                           conor_l_comp$bmi)

conor_l_comp$waist_hip_ratio <- ifelse(is.na(conor_l_comp$waist_hip_ratio),
                                    conor_imp05_MR$waist_hip_ratio,
                                    conor_l_comp$waist_hip_ratio)

#Repeat the above procedure for the training data
```

***Step IV: Fit Survival and ML models to the Training Data***

```
#Start with univariate models
library(survival)
library(ranger)
library(randomForestSRC)

conor_s_yvars <- conor_small[,c(1:3)]
conor_s_comp <- cbind(conor_s_yvars, conor_s_comp)
library(survival)
cocox_mood <- coxph(Surv(fup_months, suicide)~ mood_symps, data=conor_s_comp,
                    x=TRUE, ties="efron")
cocox_inj <- coxph(Surv(fup_months, suicide)~ injur_hosp, data=conor_s_comp,
                   x=TRUE, ties="efron")

cocox_male <- coxph(Surv(fup_months, suicide)~ male, data=conor_s_comp,
                    x=TRUE, ties="efron")

cocox_smoker_daily <- coxph(Surv(fup_months, suicide)~ smoke_daily,
                            data=conor_s_comp, x=TRUE, ties="efron")
```

```
cocox_married <- coxph(Surv(fup_months, suicide)~
                         married, data=conor_s_comp, x=TRUE, ties="efron")

cocox_spouse <- coxph(Surv(fup_months, suicide)~
                        live_w_spouse, data=conor_s_comp, x=TRUE, ties="efron")

cocox_w_hip <- coxph(Surv(fup_months, suicide)~ waist_hip_ratio, data=conor_s_comp,
                     x=TRUE, ties="efron")
cocox_low_inc <- coxph(Surv(fup_months, suicide)~ low_income_pct, data=conor_s_comp,
                       x=TRUE, ties="efron")

cocox_hrs_in_smk <- coxph(Surv(fup_months, suicide)~ hrs_in_smoke_rooms, data=conor_s_comp,
                          x=TRUE, ties="efron")
cocox_trig <- coxph(Surv(fup_months, suicide)~ trig_mmol,
                    data=conor_s_comp, x=TRUE, ties="efron")

cocox_gluc <- coxph(Surv(fup_months, suicide)~ gluc_mmol,
                    data=conor_s_comp, x=TRUE, ties="efron")

cocox_hdl <- coxph(Surv(fup_months, suicide)~
                     hdl_mmol, data=conor_s_comp, x=TRUE, ties="efron")

cocox_chol <- coxph(Surv(fup_months, suicide)~ tot_chol_mmol,
                    data=conor_s_comp, x=TRUE, ties="efron")

cocox_lt_exer <- coxph(Surv(fup_months, suicide)~
                      freq_light_act_leis, data=conor_s_comp,
                    x=TRUE, ties="efron")

cocox_yrs_smk <- coxph(Surv(fup_months, suicide)~
                         yrs_smk_frac_life, data=conor_s_comp,
                       x=TRUE, ties="efron")

cocox_hrd_exer <- coxph(Surv(fup_months, suicide)~
                         freq_hard_act_leis, data=conor_s_comp,
                       x=TRUE, ties="efron")

cocox_bp <- coxph(Surv(fup_months, suicide)~ bp_med,
                  data=conor_s_comp, x=TRUE, ties="efron")
cocox_age <- coxph(Surv(fup_months, suicide)~
                     age, data=conor_s_comp, x=TRUE, ties="efron")

cocox_month_born <- coxph(Surv(fup_months, suicide)~ month_born,
                   data=conor_s_comp, x=TRUE, ties="efron")

cocox_educ <- coxph(Surv(fup_months, suicide)~
                            yrs_educ, data=conor_s_comp, x=TRUE, ties="efron")


#Now create a survival formula for multivariable Cox models by gender.
#These variables were significant at p = .20 in the univariate models.

surv_formula_ltd<- formula(paste('Surv(', 'fup_months', ',', 'suicide', ')~',
                              'freq_hard_act_leis',
                              '+smoke_daily',
                              '+age',
                              '+live_w_spouse',
                              '+waist_hip_ratio',
```

```
                              '+low_income_pct',
                              '+mood_symps',
                              '+trig_mmol',
                              '+hrs_smoke_rooms',
                              '+married',
                              '+sec_smoke_chld',
                              '+alc_use'))

#Fit those models to males/females separately in the training set

cox_multi_females<- coxph(surv_formula_ltd, data=conor_s_females,
ties="efron", x=TRUE, y=TRUE)
cox_multi_males <-coxph(surv_formula_ltd, data=conor_s_males, ties="efron",
x=TRUE, y=TRUE))


#Test the proportional hazards assumption

males_prop_haz <- cox.zph(cox_multi_males)
females_prop_haz <-cox.zph(cox_multi_females)
###################################
#Create a Random Survival Forest Model
###################################
#Create the RSF formula
surv_formula_nosex <- formula(paste('Surv(', 'fup_months', ',', 'suicide', ') ~ ',
                              'bmi',
                              '+freq_hard_act_leis',
                              '+month_born',
                              '+injur_hosp',
                              '+smoke_daily',
                              '+age',
                              '+live_w_spouse',
                              '+waist_hip_ratio',
                              '+low_income_pct',
                              '+mood_symps',
                              '+trig_mmol',
                              '+gluc_mmol',
                              '+hdl_mmol',
                              '+hrs_smoke_rooms',
                              '+freq_light_act_leis',
                              '+married',
                              '+sec_smoke_chld',
                              '+alc_use',
                              '+yrs_smk_frac_life',
                              '+bp_med',
                              '+yrs_educ',
                              '+tot_chol_mmol'))

#First find the optimal mtry and node size,
#separately for males and females

opt_small_males <- tune(surv_formula_nosex, data= conor_s_males, trace =
TRUE, doBest = FALSE, ntreeTry = 500)
opt_small_females <- tune(surv_formula_sex, data= conor_s_females, trace =
TRUE, doBest = FALSE, ntreeTry = 500)

#Now fit separate RSFs for males and females
conor_rsf_males <-rfsrc(surv_formula_nosex,
                          data = conor_s_males,
```

```
                        ntree = 500,
                        nodesize=6,
                        nsplit=10,
                        mtry=4,
                        samptype="swor",
                        splitrule = "logrank",
                        importance = "none",
                        block.size = "NULL",
                        var.used = FALSE,
                        do.trace=10,
                        seed=-13)

conor_rsf_females <-rfsrc(surv_formula_nosex,
                        data = conor_s_females,
                        ntree = 500,
                        nodesize=6,
                        nsplit=10,
                        mtry=4,
                        samptype="swor",
                        splitrule = "logrank",
                        importance = "none",
                        block.size = "NULL",
                        var.used = FALSE,
                        do.trace=10,
                        seed=-13)
```

***Step V: Inspect the Top Predictors***

```
#For the Cox models, simply examine the variables with significant p values
#For the RSFs: first refit the RSF model above using the ranger package
#then call the importance_pvalues function

#####This is the equivalent of the RSF model
library(ranger)
conor_rgr_males <- ranger(formula = NULL,
                          data = conor_s_males,
                          dependent.variable.name = "fup_months",
                          status.variable.name = "suicide",
                          num.trees = 500,
                          respect.unordered.factors = "ignore",
                          mtry=4,
                          min.node.size = 6,
                          importance = "impurity_corrected",
                          num.random.splits = 10,
                          splitrule = "extratrees",
                          alpha = .05,
                          write.forest = TRUE,
                          replace = FALSE,
                          oob.error = TRUE,
                          verbose = TRUE,
                          seed=-13)

conor_rgr_females <- ranger(formula = NULL,
                          data = conor_s_females,
                          dependent.variable.name = "fup_months",
                          status.variable.name = "suicide",
                          num.trees = 500,
                          respect.unordered.factors = "ignore",
                          mtry=4,
                          min.node.size = 6,
```

```
                          importance = "impurity_corrected",
                          num.random.splits = 10,
                          splitrule = "extratrees",
                          alpha = .05,
                          write.forest = TRUE,
                          replace = FALSE,
                          oob.error = TRUE,
                          verbose = TRUE,
                          seed=-13)

rgr_vimp_alt <- importance_pvalues(conor_model_rgr,
                              formula = surv_formula,
                              data=conor_s_comp_anon,
                              method= "altmann",
                              num.permutations = 500)

rgr_vimp_alt_males <- importance_pvalues(conor_rgr_males,
                                    formula = surv_formula_nosex,
                                    data = conor_s_males,
                                    method= "altmann",
                                    num.permutations = 500)

rgr_vimp_alt_females <- importance_pvalues(conor_rgr_females,
                                formula = surv_formula_nosex,
                                data= conor_s_females,
                                method= "altmann",
                                num.permutations = 500)
```

***Step VI-VII: Predict the Outcomes in the Test Set and Assess Accuracy***

```
#Cox predictions here
pred_large_cox_males_CONOR <- predict(cox_multi_males,
                                      newdata = conor_l_males,
                                      type="lp")
pred_large_cox_females_CONOR <- predict(cox_multi_females,
                                      newdata = conor_l_females,
                                      type="lp")

#Random Survival Forest predictions
pred_large_rsf_males_CONOR <- predict(conor_rsf_males,
                                      newdata = conor_l_males,
                                      importance = "none")

pred_large_rsf_females_CONOR <- predict(conor_rsf_females,
                                      newdata = conor_l_females,
                                      importance = "none")
###Assess the ROC of predictions at each quartile of follow-up time#####
#####This is done using the Stata program stroccurve

#Extract the markers for the RSF objects
#These need to be exported to Stata using the haven package
pred_rsf_male_marker <- pred_large_rsf_males_CONOR$predicted
pred_rsf_female_marker <- pred_large_rsf_females_CONOR$predicted
pred_cox_male_marker <- pred_large_cox_males_CONOR
pred_cox_female_marker <-pred_large_cox_females_CONOR

#################The code below is now in Stata##############
net install stroccurve
#import the following objects from R: conor_l_males, conor_l_females, pred_rsf_male_marker,
#pred_rsf_female_marker,pred_cox_male_marker,pred_cox_female_marker
```

```
stset fup_months, failure(suicide) id(id)
//MALES
//cox models
stroccurve pred_rsf_male_marker, timepoint(181) nearest
stroccurve pred_rsf_male_marker, timepoint(212) nearest
stroccurve pred_rsf_male_marker, timepoint(243) nearest
stroccurve pred_rsf_male_marker, timepoint(267) nearest

//rsf models
stroccurve pred_cox_male_marker, timepoint(181) nearest
stroccurve pred_cox_male_marker, timepoint(212) nearest
stroccurve pred_cox_male_marker, timepoint(243) nearest
stroccurve pred_cox_male_marker, timepoint(267) nearest

//FEMALES
//cox models
stroccurve pred_rsf_female_marker, timepoint(181) nearest
stroccurve pred_rsf_female_marker, timepoint(212) nearest
stroccurve pred_rsf_female_marker, timepoint(243) nearest
stroccurve pred_rsf_female_marker, timepoint(267) nearest

//rsf models
stroccurve pred_cox_female_marker, timepoint(181) nearest
stroccurve pred_cox_female_marker, timepoint(212) nearest
stroccurve pred_cox_female_marker, timepoint(243) nearest
stroccurve pred_cox_female_marker, timepoint(267) nearest
##############End of Stata Code################################
```

### Saskatoon Clinical Sample

#### *Steps I & II: Partitioning and balancing*

```
library(caret)
set.seed(66)
tr_index <-createDataPartition(sk_anon$Ysuic_, p=0.85, list=FALSE,
            times=1)

sk_trn_df <- sk_anon[tr_index,]
sk_tst_df <- sk_anon[-tr_index,]

#the training file needs to be contain equal numbers of #suicides/non-suicides
```

#### *Step III. Imputation*

This was not performed because the variables with missing values had very high missing rates.

#### *Step IV: Fit Survival and ML models to the Training Data*

```
#######Discrete Survival Models#######

#fit a baseline time only model
sk_baseline <- glm(formula = Ysuic_ ~ interval, family=binomial(link =
"logit"),data = sk_trn_df)
#Add age variable
sk_baseline_age_ <- glm(formula = Ysuic_ ~ interval + age_at_index,
                        family=binomial(link = "logit"),
                        data = sk_trn_df)


#None of the following models were estimable:
sk_baseline_male <- glm(formula = Ysuic_ ~ interval + male,
                        family=binomial(link = "logit"),
                        data = sk_trn_df)
```

```
sk_baseline_RESH <- glm(formula = Ysuic_ ~ interval + RESH,
                        family=binomial(link = "logit"),
                        data = sk_trn_df)

sk_baseline_Ysh <- glm(formula = Ysuic_ ~ interval + Ysh_,
                        family=binomial(link = "logit"),
                        data = sk_trn_df)
sk_baseline_AMIS <- glm(formula = Ysuic_ ~ interval +
                       num_prior_AMIS_visits,
                       family=binomial(link = "logit"),
                       data = sk_trn_df)

sk_baseline_subst <- glm(formula = Ysuic_ ~ interval +
                        family=binomial(link = "logit"),
                        data = sk_trn_df)
sk_baseline_dep <- glm(formula = Ysuic_ ~ interval + depcombined_,
                         family=binomial(link = "logit"),
                         data = sk_trn_df)
sk_baseline_eat <- glm(formula = Ysuic_ ~ interval + eatingcombined_,
                       family=binomial(link = "logit"),
                       data = sk_trn_df)

sk_baseline_adhd <- glm(formula = Ysuic_ ~ interval + adhdcombined_,
                       family=binomial(link = "logit"),
                       data = sk_trn_df)
sk_baseline_person <- glm(formula = Ysuic_ ~ interval +
                       personcombined_,
                        family=binomial(link = "logit"),
                        data = sk_trn_df)

sk_baseline_anx <- glm(formula = Ysuic_ ~ interval + anxcombined_,
                          family=binomial(link = "logit"),
                          data = sk_trn_df)

#############Historical Random Forest Model#############
library(htree)

historical_predictors=
  match(c("RESH", "Ysh_","anxcombined_", "depcombined_",
        "substcombined_", "schizcombined_",
        "maniacombined_", "adhdcombined_",
        "num_prior_AMIS_visits"),names(sk_trn_df))

concurrent_predictors=(match(c("male","age_at_index"),
                             names(sk_trn_df)))

control=list(vh=historical_predictors,
             vc=concurrent_predictors,
             nodesize=13,
             se=FALSE,
             method="freq",
             ntrees=200,
             delta=2,
             mtry=1,
             sample_fraction=0.2)

hrf_model_sk <- hrf(x=sk_trn_df, id=sk_trn_df$fake_id,
                    time=sk_trn_df$interval,
                    yindx=4,
```

```
                    control=control,
                    classify=FALSE)
```

***Step V: Inspect the Top Predictors***

```
#For the discrete survival model, only age was the significant predictor.
###examine variable importance for the historical random forest model
hrf_vimp_train <- varimp_hrf(hrf_model_sk, nperm = 20, parallel =
                             TRUE)
```

***Step VI-VII: Predict the Outcomes in the Test Set and Assess Accuracy***

```
#Discrete survival model
sk_logistic_predict <- predict(sk_baseline_age_,
                            newdata = sk_tst_df,
                            type="response")

#Historical random forest
hrf_test_pred <- predict_hrf(object=hrf_model_sk, x=sk_tst_df,
                            id=sk_tst_df$fake_id, se=FALSE)

###Assess the ROC of predictions at each interval that has at least
###1 suicide death in the test set.

#Export the following objects to Stata using the haven package:
#sk_tst_df, sk_logistic_predict, hrf_test_pred

##################The code below is now in Stata###############
stset interval, failure(Ysuic_) id(fake_id) origin(entry)


//Logistic regression models
stroccurve sk_logistic_predict, timepoint(183) nearest
stroccurve sk_logistic_predict, timepoint(366) nearest
stroccurve sk_logistic_predict, timepoint(549) nearest
stroccurve sk_logistic_predict, timepoint(915) nearest
stroccurve sk_logistic_predict, timepoint(1098) nearest

//Historical Random Forest
stroccurve hrf_test_pred, timepoint(183) nearest
stroccurve hrf_test_pred, timepoint(183) nearest
stroccurve hrf_test_pred, timepoint(183) nearest
stroccurve hrf_test_pred, timepoint(183) nearest
stroccurve hrf_test_pred, timepoint(183) nearest

##############End of Stata Code################################
```

## REFERENCES


1. R. O'Brien and H. Ishwaran, "A random forests quantile classifier for class imbalanced data," Pattern recognition **90**, 232–249 (2019).
2. S. G. Liao, Y. Lin, D. D. Kang, D. Chandra, J. Bon, N. Kaminski, F. C. Sciurba, and G. C. Tseng, "Missing value imputation in high-dimensional phenomic data: imputable or not, and how?" BMC bioinformatics **15**, 1–12 (2014).
3. D. J. Stekhoven and P. Bühlmann, "Missforest—non-parametric missing value imputation for mixed-type data," Bioinformatics **28**, 112–118 (2012).
4. V. Audigier, F. Husson, and J. Josse, "A principal component method to impute missing values for mixed data," Adv. Data Analysis Classif. **10**, 5–26 (2016).
5. L. Gaudette and N. Japkowicz, "Evaluation methods for ordinal classification," in *Canadian conference on artificial intelligence,* (Springer, 2009), pp. 207–210.
6. E. M. Peters, A. John, R. Bowen, M. Baetz, and L. Balbuena, "Neuroticism and suicide in

a general population cohort: results from the uk biobank project," BJPsych open **4**, 62–68 (2018).
7. L. Balbuena and R. Tempier, "Independent association of chronic smoking and abstinence with suicide," Psychiatr. services **66**, 186–192 (2015).
8. H. Ishwaran, U. B. Kogalur, and M. U. B. Kogalur, "Package 'randomforestsrc'," (2021).
9. A. Altmann, L. Toloşi, O. Sander, and T. Lengauer, "Permutation importance: a corrected feature importance measure," Bioinformatics **26**, 1340–1347 (2010).
10. M. N. Wright and A. Ziegler, "ranger: A fast implementation of random forests for high dimensional data in c++ and r," arXiv preprint arXiv:1508.04409 (2015).
11. P. J. Heagerty, T. Lumley, and M. S. Pepe, "Time-dependent roc curves for censored survival data and a diagnostic marker," Biometrics **56**, 337–344 (2000).
12. N. J. Perkins and E. F. Schisterman, "The inconsistency of "optimal" cutpoints obtained using two criteria based on the receiver operating characteristic curve," Am. journal epidemiology **163**, 670–675 (2006).
13. M. Cattaneo, P. Malighetti, and D. Spinelli, "Estimating receiver operative characteristic curves for time-dependent outcomes: The stroccurve package," The Stata J. **17**, 1015–1023 (2017).
14. J. Sexton, "Package 'htree: Historical tree ensembles for longitudinal data'," (2018).
15. T. Dankowski and A. Ziegler, "Calibrating random forests for probability estimation," Stat. medicine **35**, 3949–3960 (2016).
16. A. Bansal and P. J. Heagerty, "A tutorial on evaluating the time-varying discrimination accuracy of survival models used in dynamic decision making," Med. Decis. Mak. **38**, 904–916 (2018).
